# Evaluation of Suspected Achilles Tendon Rupture Managed Through Virtual Fracture Clinic Pathway in Busy Major Trauma Centre

**DOI:** 10.7759/cureus.93309

**Published:** 2025-09-26

**Authors:** Masroor Ahmed, Khaled Ibrahim, Mohammad J Faisal, Mayank Kumar, Ahmad W Mohamed, Prerana Gogoi, Matija Krkovic

**Affiliations:** 1 Trauma and Orthopaedics, Cambridge University Hospitals NHS Foundation Trust, Cambridge, GBR; 2 Surgery, Cambridge University Hospitals NHS Foundation Trust, Cambridge, GBR

**Keywords:** achilles tendon rupture, conservative, management, re-ruptures, surgical repair, virtual fracture clinic

## Abstract

Introduction

Achilles tendon rupture is one of the most common tendon injuries, affecting both males and females at a young age in athletic populations. These injuries can be best managed through the virtual fracture clinic once these patients present in the emergency department with suspected Achilles tendon ruptures. This study aims to evaluate the management of patients with Achilles tendon ruptures who are referred to virtual fracture clinics in the emergency department of a busy major trauma centre.

Materials and methods

This retrospective study reviewed all the patients referred from the emergency department to the virtual fracture clinic with suspected Achilles tendon ruptures. Data were collected through the electronic medical record system and by reviewing the patient's notes, which were analysed using SPSS Statistics version 20.0 (IBM Corp., Armonk, NY, USA). Data were reviewed in terms of age, gender, side affected, ultrasound findings for full tear or partial tear of Achilles tendon, gastrocnemius tear, chronic tendinosis, and no tear of Achilles tendon. Data were reviewed in terms of management of the injury, whether conservative management or surgical management was adopted as a treatment option.

Results

A total of 170 patients were identified through the electronic records. In 10 patients, no ultrasound studies were performed, and three patients did not attend; therefore, they were excluded from the study according to our exclusion criteria. The patient's mean age in our study was 46.69 years. Out of 157 patients, 119 (75.8%) were male and 38 (24.2%) were female. The right side was involved in 89 (56.7%) of cases, and the left side was involved in 68 (43.3%). According to the ultrasound results, 94 patients (59.9%) had a complete tear of the Achilles tendon, 41 patients (26.1%) had a partial tear, two patients (1.3%) had chronic tendonitis, three patients (1.9%) had a gastrocnemius tear, and 17 patients (10.8%) showed no tears or problems.

Conclusion

The suspected Achilles tendon ruptures can be effectively managed through the virtual fracture clinic after referral from the emergency department. This decreases the chances of missing any injuries and is found to be safe, effective, and reproducible.

## Introduction

Virtual fracture clinics, now operational in most NHS trusts, have proven to be a safe, effective, and cost-saving solution for managing orthopaedic injuries. These clinics have significantly reduced the workload of standard fracture clinics and the associated costs [1,2].

The Achilles tendon is one of the strongest tendons found in the human body [3]. It arises from the fibres of the gastrocnemius and soleus muscles in the posterior compartment of the lower leg and inserts at the posterior calcaneal tuberosity [4]. Tendo-Achilles rupture is one of the most common orthopaedic presentations, with an incidence of 40/100,000 persons affected annually [5]. A Tendo-Achilles rupture usually happens in the middle part of the tendon, about 2 to 6 cm from where it attaches to the calcaneum, mainly because this area has poor blood supply [6].

Accurate diagnosis of Achilles tendon injuries is crucial, as they are among the most commonly missed orthopaedic injuries, accounting for about 20% of cases [7]. The difficulty is that even after the injury, patients can still move around, and an organised haematoma at the rupture site can make it challenging to check for a noticeable gap in the tendon [8,9].

Management of Achilles tendon rupture has long been a topic of controversy; both operative and non-operative treatment options have been considered optimal for managing Achilles tendon ruptures.

This study aims to evaluate the management of patients with suspected Achilles tendon ruptures who are referred to virtual fracture clinics in the emergency department of a busy major trauma centre.

## Materials and methods

This retrospective observational study was conducted in the Trauma and Orthopaedics Department at Cambridge University Hospitals NHS Foundation Trust, involving patients referred from the emergency department with suspected Achilles tendon ruptures between January 2023 and December 2024. Data were collected after approval from the Cambridge University Hospital NHS Foundation Trust audit and research committee (clinical project approval number PRN 13117).

Data were extracted from the hospital IT records for patients referred for suspected Achilles tendon ruptures and their notes. Data were collected on age, gender, laterality, ultrasound findings, and patient management, specifically whether the management was conservative or surgical. Patients were assessed for re-ruptures.

We included all the patients referred from the emergency department to the virtual fracture clinic with suspicion of closed Achilles tendon ruptures. We excluded patients with open ruptures, missing data, and those with clinical diagnoses who did not have an ultrasound performed from the study. Patients were reviewed in the virtual fracture clinic, and after review, they were either booked into the specialist foot and ankle clinic or any lower limb clinic. 

Descriptive statistics illustrated the demographics, including gender, laterality regarding the affected side, and injuries sustained as per ultrasound findings. The chi-square test assessed the association between re-rupture of the Achilles tendon and Achilles tendon rupture (full rupture/partial rupture) as well as gender. The strength of association was quantified using Cramer's V, with a significance threshold set at a p-value of 0.05. SPSS Statistics for Windows version 20.0 (IBM Corp., Armonk, NY, USA) was utilised for data analysis.

## Results

A total of 170 patients were identified through the electronic records, who were referred to the virtual fracture clinic after presentation and assessment in the emergency department with suspected Achilles tendon ruptures. In 10 patients, no ultrasound studies were performed, and three patients did not attend; therefore, they were excluded from the study according to our exclusion criteria. The mean age of the patients in our study was 46.69 years, with a range of 16-85 years. Out of 157 patients, 119 (75.8%) were male and 38 (24.2%) were female.

**Figure 1:**
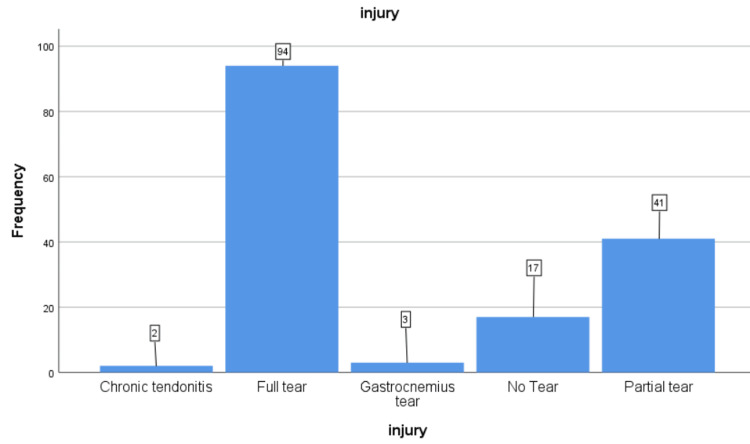
Injury findings based on ultrasound.

In terms of laterality, the right side was involved in 89 (56.7%) of cases, and the left side was involved in 68 (43.3%) of cases. According to the ultrasound results, 94 (59.9%) patients had a complete tear of the Achilles tendon, 41 (26.1%) patients had a partial tear, two patients (1.3%) had chronic tendonitis, three patients (1.9%) had a gastrocnemius tear, and 17 patients (10.8%) showed no tears or problems, as shown in Figure 1.

Patients with partial rupture were managed conservatively; out of 41 patients with partial rupture, one patient sustained re-rupture of the Achilles tendon. Out of 94 patients with a full tear of the Achilles tendon, five patients underwent surgical treatment, and 89 patients were managed non-operatively. None of the patients who underwent surgery developed re-rupture of the Achilles tendon; however, 3 (3.3%) out of 89 patients managed conservatively developed re-rupture of the Achilles tendon. In total, 4 (3.07%) out of the 130 patients who were managed non-operatively sustained a re-rupture of the Achilles tendon.

In our study, a chi-square test demonstrated no significant association of re-rupture with Achilles tendon rupture (partial and full tears) and gender (p < 0.81, Φ = 0.20), as shown in Table 1. 

**Table 1:** Association of re-rupture with Achilles tendon rupture (full tear/partial tear) and gender.

Gender	Re-rupture	Full tear	Partial tear	Total	χ²	p-value
Female	No	20	9	29	0.56	0.813
	Yes	1	0	1		
Male	No	71	31	102		
	Yes	2	1	3		
Total	No	91	40	131		
	Yes	3	1	4		

## Discussion

The treatment strategy for Achilles tendon ruptures has undergone significant changes, including a shift in momentum from operative to non-operative treatment and a standardised rehabilitation protocol focused on early mobilisation. The primary aim in managing Achilles tendon rupture is to promote healing and return to normal activities. Several studies have suggested that reducing the duration of immobilisation and implementing effective rehabilitation can reduce the risk of Achilles tendon rupture [10, 11]. Initially, operative repair for Achilles tendon ruptures was considered the better option due to a reduced risk of re-rupture. Our trust has established a standard management system for treating these injuries. Once the patient is referred to the virtual fracture clinic, they are placed in an Air Cast boot or black boot with three wedges and allowed to bear full weight. The clinic reviews the patient in four weeks and starts to remove one wedge per week, remaining in the flat boot for one week. Patients are then reviewed in eight weeks in the clinic, where, after a clinical evaluation of the skin and tendon integrity, the boot is removed and the patient is discharged to physiotherapy.

A study conducted by Lantto et al. advocated for surgical repair, as it can lead to an earlier recovery in terms of calf muscle strength and ankle joint range of motion [12]. Surgery, despite its advantages, also has drawbacks, particularly complications such as wound infections, scar adhesions, tendon necrosis, and nerve injuries, which can be disastrous for the patient [10,13]. Early weight-bearing has been beneficial for patients with Achilles tendon injuries, as loading increases the maturation of collagen fibres, leading to tendon healing [14,15]. It is also beneficial in preventing muscle atrophy associated with non-weight-bearing activities [16].

In our study, 119 (75.8%) patients who experienced injury were males. This finding aligns with Noback et al., who showed that the majority of patients affected by Achilles tendon rupture are male, as was the case in our study [17]. In our study, we observed that patients who sustained partial- and full-thickness tears and were managed conservatively, only four patients presented with re-ruptures, a re-rupture rate of only 3.07%, which is comparable to the study conducted by Robertson et al. [18].

Our study has demonstrated that managing Achilles tendon ruptures through the virtual fracture clinic is a highly reliable, effective, and reproducible approach. A standard treatment plan is available, ensuring consistency for every surgeon involved in managing these patients. Especially when managing these injuries in an Air Cast boot with wedges and full weight-bearing, this approach has provided favourable results.

This study has some limitations; being a retrospective observational study, we do not have functional outcome assessments for this injury, and there is no calculation of patient satisfaction during the entire treatment duration. We will be recommending managing the Achilles tendon ruptures through the virtual clinic fracture pathway based on the experience in our institute.

## Conclusions

Achilles tendon injury is one of the commonest injuries being reviewed in the emergency department and referred to orthopaedic surgeons. The suspected Achilles tendon ruptures can be effectively managed through the virtual fracture clinic after referral from the emergency department. This protocol decreases the chances of missing any injuries and is found to be safe, effective, and reproducible.
